# Acceleration of Translational Mesenchymal Stromal Cell Therapy Through Consistent Quality GMP Manufacturing

**DOI:** 10.3389/fcell.2021.648472

**Published:** 2021-04-13

**Authors:** Premkumar Jayaraman, Ryan Lim, Jacqueline Ng, Mohan C. Vemuri

**Affiliations:** ^1^Thermo Fisher Scientific, Singapore, Singapore; ^2^Thermo Fisher Scientific, Frederick, MD, United States

**Keywords:** mesenchymal stromal cells, cell therapy, GMP manufacturing, closed and automation systems, characterization and potency, regulatory compliance

## Abstract

Human mesenchymal stromal cell (hMSC) therapy has been gaining immense interest in regenerative medicine and quite recently for its immunomodulatory properties in COVID-19 treatment. Currently, the use of hMSCs for various diseases is being investigated in >900 clinical trials. Despite the huge effort, setting up consistent and robust scalable manufacturing to meet regulatory compliance across various global regions remains a nagging challenge. This is in part due to a lack of definitive consensus for quality control checkpoint assays starting from cell isolation to expansion and final release criterion of clinical grade hMSCs. In this review, we highlight the bottlenecks associated with hMSC-based therapies and propose solutions for consistent GMP manufacturing of hMSCs starting from raw materials selection, closed and modular systems of manufacturing, characterization, functional testing, quality control, and safety testing for release criteria. We also discuss the standard regulatory compliances adopted by current clinical trials to broaden our view on the expectations across different jurisdictions worldwide.

## Introduction

The immense potential of human mesenchymal stromal cells (hMSCs) for regenerative capacity and immunosuppression has been increasingly explored for treating a diverse group of diseases such as neurodegenerative, cardiovascular, autoimmune, bone, cartilage, kidney, liver, cancer, and other disorders (Galipeau and Sensébé, 2018; Pittenger et al., 2019; Saeedi et al., 2019; Kabat et al., 2020). It is well documented now that hMSCs, after transplantation, exert the therapeutic effects through two mechanisms: (i) differentiate into functional cells and facilitate tissue repair by homing into the injured sites (Mastrolia et al., 2019) and (ii) secrete growth factors and cytokines to stimulate immunosuppressive effects by modulating the immune cells (T-cells, dendritic cells, NK cells, and B-cells), angiogenesis, and extracellular matrix remodeling (Pittenger et al., 2019). In addition, hMSCs have low immunogenicity and thus they have the potential to be used for both autologous and allogeneic therapy (Hassouna et al., 2019).

With over 900 hMSC clinical trials listed on ClinicalTrials.gov, the field has expanded its understanding and application of hMSCs and seems poised for success (Levy et al., 2020). Initial successes include the 2018 European approval of TiGenix/Takeda, Alofisel^®^, for complex perianal fistulas in Crohn’s disease. However, only around 300 trials were completed as of 2020, and the total number of approved hMSCs therapy stands at just 10 (Levy et al., 2020). Earlier this year, one of the approved hMSC products’ remestemcel-L (Ryoncil^TM^, Mesoblast) phase 3 clinical trial showed significant improvement in pediatric patients who failed to respond to steroid treatment for acute graft-versus-host disease (aGVHD) (Kurtzberg et al., 2020).

Given the backdrop of increasing interest in using hMSC therapies to fulfill unmet patient needs, there are still inherent industry challenges that would pose barriers to market access, especially within the manufacturing process. Some of the main challenges are product consistency in terms of quality and efficacy, raw material qualification to ensure that the clinical product meets regulatory compliance, cost of cell processing as manufacturing is scaled up or scaled out, and lack of advanced check-point analytical tools to carry out the process and product quality assessment. If the clinical product is not consistent, batch failures are imminent, leading to loss of productivity and compromised sustainability. Protocol amendments can impact the timely progress of all functions including R&D, process development, quality control, manufacturing, regulatory, and clinical testing. To implement a substantial clinical protocol amendment, the median costs can be $141,000 for phase II and $535,000 for phase III protocols (Getz et al., 2016). That is why choosing the right starting raw materials is very important as early as the process development stage. When moving toward clinical trials, developers need more safety and regulatory features to foresee and meet regulatory requirements. It is the responsibility of the hMSC manufacturer to qualify the performance of the raw materials, assess the lot-to-lot variability, test residuals on the final cell product, and determine the need for any additional safety testing. Furthermore, the manufacturer needs to qualify whether the intended suppliers can provide raw material traceability, characterization, and regulatory filing support documentation.

Concomitantly, both autologous and allogeneic cell therapy manufacturing workflow comprises of many different unit operations and, thus, is very complex and labor-intensive due to open processing. Open manipulations are prone to errors and contamination leading to a risk of failed production runs. Besides, manual methods to synchronize different steps in scale-out or scale-up processes and proper workflow documentation to satisfy GMP compliance adds another layer of complexity. In contrast, closing the process and automating the entire manufacturing workflow through digital integration would reduce the risks of open operations and improves product consistency, which is a critical necessity for a GMP setting (Moutsatsou et al., 2019).

Furthermore, identification and assessment of critical quality attributes (purity, potency, and safety) for release criteria as early as the process development stage would ensure product consistency during commercial manufacturing (National Academies of Sciences Engineering, Medicine, Health and Medicine Division, Board on Health Sciences Policy, and Forum on Regenerative Medicine, 2017). In 2006, the International Society of Cell and Gene Therapy (ISCT) came up with a “minimal criteria” for defining hMSC (Dominici et al., 2006). While it is useful in defining the identity and some functionality of hMSCs, it stopped short of defining other critical attributes such as its immunomodulatory capability of cells and other novel biomarkers (Samsonraj et al., 2017). In 2019, the criteria were updated by the ISCT to include the tissue-source origin of the cells and a matrix of functional assays such as secretion of trophic factors ensuring more meaningful information is collected to properly assess the therapeutic potential of hMSCs (Viswanathan et al., 2019). Yet, there remains a lack of a minimum set of standard guide release criteria that hMSC manufacturers targeting different diseases can adopt for regulatory approvals. Additionally, if the manufacturers are targeting their hMSC products for multiple regions, it is important to align with each region’s regulatory guidance as it is imperative to note that each region has unique raw material regulatory guidance documentation.

In this review, we discuss the raw materials considerations for hMSC manufacturing with a particular focus on QC/safety testing expectations from global regulatory guidelines. We propose a rationale of why closed, automated, and modular systems are integral to GMP manufacturing and discuss possible workflow solutions for both scale-out and scale-up processes. We focus on hMSC product characterization tools and provide insights to improve existing assays throughout the development and manufacturing process. We also shed light on the regulatory perspectives of current hMSC products in the market and potential future guidelines for regulatory approvals in different global regions.

## Consideration in the Selection and Qualification of Culture Systems Used in hMsc Manufacturing

hMSC culture systems have evolved over the last 40 years. Table 1 summarizes common hMSC culture systems and critical raw materials used following regulatory expectations of quality and safety testing. There are many do-it-yourself and commercially available serum-free and xeno-free (XF) culture systems currently; however, there is no harmonization in the way culture systems are being classified. To avoid ambiguity, we attempt to define the classification of culture media in Table 1 (Jayme and Smith, 2000). ISO/TS 20399 also lists definitions of ancillary materials that the field could aim to adopt. Today, there are over 30 hMSC expansion media marketed as “XF” by commercial suppliers (Gottipamula et al., 2013). We will focus on discussing XF culture systems supplemented with human platelet lysate (hPL) or recombinant human proteins and growth factors as these are current trends in the field (Guiotto et al., 2020; Yan et al., 2020).

**TABLE 1:** Classification of hMSC culture systems.

Classification	Definition
Serum-containing media	Contains animal or human serum (i.e., FBS or Human serum)
Serum-free media	Does not contain animal or human serum or plasma as direct/primary ingredients. Media may still contain proteins purified from the blood (i.e., BSA and HSA)
Xeno-Free media	Contains human-derived blood components as direct ingredients (i.e., hPL, human serum) and may contain human proteins purified from human blood (i.e., HSA) and human recombinant growth factors.
Animal origin-free	Does not contain any human or animal components at the product and process level. Does not contain human recombinant proteins and growth factors. Could contain biological proteins expressed in plant and rice (i.e., soy hydrolyzate)
Chemically defined media	Media formulation with known chemical components and structures. Does not contain any proteins or complex raw materials.

Friedenstein et al. (1970) were the first to report the culture of fibroblast-like colonies from guinea pig bone marrow in media supplemented with fetal bovine serum (FBS). Media supplemented with 10–20% FBS has since been recognized as a conventional method to expand hMSCs from various tissue sources (Haynesworth et al., 1992) and has been used as an ancillary reagent in clinical trials since the early 1990s.

Safety concerns using undefined animal serum include risks of introducing pathogens, exposing patients to xenogenic infections, and unintended immunological reactions to bovine proteins (Macy et al., 1989; Heiskanen et al., 2007). Twenty to fifty percent of commercial FBS is tested positive for viruses and not all lots are suitable for MSC isolation and expansion (Wessman and Levings, 1999; Gottipamula et al., 2013). Although regulatory authorities allow the use of FBS as a raw material for clinical production, cell therapy manufacturers would have to ensure that FBS is adequately controlled and that viral testing/inactivation processes (gamma-irradiation/mycoplasma/sterility, 9CFR virus testing) (TSE/BSE sourcing) and specific risk assessments are thoroughly performed in conformity to the relevant regulatory guidance (Supplementary Table 1).

Given the lot-to-lot variability of FBS, significant investment in time and costs have to be made in rigorous screening, selection, and validation of suitable lots to ensure consistency and reproducibility in culture performance expansion (van der Valk et al., 2004). The presence of FBS during the hMSC expansion could also influence cell quality attributes—hMSC cultures could undergo early senescence with progressive loss of differentiation capacity (Bieback et al., 2012). Overall, FBS is viewed as a high-risk material (USP < 1043 > ancillary material risk tier 4) and regulatory agencies have recommended manufacturers to use non-animal, non-ruminant materials if the option exists. The field has thus shifted toward adopting xeno- and serum-free culture systems for hMSC manufacturing.

Today, hPL has been suggested as an XF substitute of FBS (Doucet et al., 2005), and it has been increasingly used in trials. A survey of bone marrow transplantation centers in Europe reported 77% of centers use hPL-supplemented media for trials utilizing hMSCs. Initially described by Doucet et al. (2005), hPL is derived from platelet-rich plasma of whole blood donations or apheresis collections (Schallmoser and Strunk, 2013). Platelets are subjected to lysis through repeated freeze/thaw cycles resulting in the release of bioactive molecules and growth factors involved in stimulating mitogenesis and promoting cell adherence (Guiotto et al., 2020; Yan et al., 2020). Research has shown that hMSCs expanded in hPL-supplemented media retain their *in vitro* and *in vivo* characteristics and generally achieve superior proliferation rates over hMSCs expanded in FBS-based systems (Schallmoser et al., 2007; Bieback et al., 2009; Ben Azouna et al., 2012; Griffiths et al., 2013). As such, hPL culture systems are viewed as a desirable option to enable large-scale commercial manufacturing of hMSCs in both 2D and 3D suspension-based platforms. There are, however, ongoing challenges with using hPL in hMSC manufacturing. hPL is undefined and its composition is inherently heterogeneous. Many factors such as donor differences (i.e., gender, age, blood group, metabolites) and production processes influence batch-to-batch variation (Lohmann et al., 2012; Pierce et al., 2017).

A key gap lies in the lack of standardized methods used in sourcing, producing, and quality/safety testing of hPL. Usually, hPL is prepared from a large allogenic pool of blood donation to balance out variation in growth factor concentrations across donors and manufactured lots. However, the size of the donor pool has recently come under regulatory scrutiny due to concerns over risks of transmitting bloodborne pathogens. European Pharmacopeia general chapter 5.2.12 recommends that pooled donations must be limited otherwise pathogen reduction treatment (PRT) needs to be applied during hPL production. Pathogens can be reduced or inactivated by several methods such as gamma-irradiation and treatment with amotosalen + ultraviolet (UV)A light, riboflavin + UVB light, UVC light, or solvent/detergent (S/D). While global regulatory agencies and pharmacopeia recommend limiting the size of the donor pool, no specific guidelines have been given aside from German regulations that restrict the size to 16 donors without the need for pathogen reduction.

hMSC therapy developers using hPL-based culture systems will face some limitations around batch consistency, safety, costs associated with outsourcing PRT, and additional performance testing to ensure that release criteria are met using hPL subjected to PRT. It is no surprise that the industry is in favor of using defined serum-free and XF formulations containing only human proteins and growth factors, but there are still several barriers to regulatory acceptance and commercialization to consider. Firstly, suppliers of hMSC XF systems do not often state that their formulation fully utilizes human recombinant proteins and growth factors. Such systems could still contain proteins purified directly from human plasma (i.e., human serum albumin or human transferrin). Cell therapy developers will have to ensure that the human plasma-derived proteins are sourced from low-risk origins and accredited blood banks and have the necessary adventitious agent testing and inactivation performed. Secondly, human recombinant proteins used in hMSC XF culture systems could be expressed in mammalian cell lines such as CHO or HEK. Regulatory guidance suggests following the principles of ICHQ5A (viral testing evaluation) and ICHQ5D (characterization/lineage history) in establishing master cell banks (MCB) for products derived from cell lines of human or animal origin. hMSC developers should consult with their suppliers on the quality and documentation available for proteins derived from mammalian MCBs. As a safer alternative, hMSC developers and suppliers could aim to use recombinant proteins expressed only in non-mammalian and non-animal cell lines. Thirdly, unlike serum and undefined hPL, hMSC XF culture systems containing purified and/or recombinant proteins lack extracellular matrix proteins to support cell adhesion. Pre-coating surfaces with common cell adhesion proteins such as human collagen and human fibronectin are required. To ease the expansion process, hMSC developers have been exploring coating-free methods by simply supplementing cell adhesion proteins directly to the culture media. However, obtaining a consistent and affordable supply of GMP-grade collagen or fibronectin and their respective animal-origin free recombinant alternatives remains a current industry challenge. Recent studies have reported the use of recombinant vitronectin for hMSC expansion as an alternative substrate to fibronectin and collagen. Recombinant vitronectin protein fragments are widely used in the expansion of pluripotent stem cells with GMP-grade, animal-origin free versions available by several commercial suppliers.

With this background, developers need to identify and evaluate regulatory compliant hMSC raw materials and optimize expansion and characteristics in small scale during the early process development stage. This exercise would serve as a prerequisite for the next stage of large-scale GMP manufacturing in a closed and automated manner.

## GMP Based Automated, Closed System Manufacturing

In this section, we will discuss the benefits of closing the process, automation, and single-use technologies (SUTs) followed by a review of existing and proposed closed automation solutions for each of the hMSC manufacturing unit operations.

MSC-based therapies require large-scale manufacturing, conserving both the phenotypic characteristics and functional potency of the donor-derived MSCs. Typically, both allogeneic and autologous hMSC manufacturing processes include cell isolation, followed by *ex vivo* cell expansion, harvesting the expanded hMSCs, wash and concentrate cells, and final fill and finish cell doses (formulation) either for direct infusion or for cryopreservation. Currently, most of these unit operations in the hMSC manufacturing are manual or semi-automated involving largely open processes (Timmins et al., 2012; Nguyen, 2016). Consequently, these are laborious, labor-intensive, prone to cross-contamination resulting in production loss, batch-to-batch inconsistency, high manufacturing costs due to the requirement of a large footprint of the facility with dedicated Class B processing areas, and increased environmental monitoring (Moutsatsou et al., 2019). This creates a major challenge for hMSC manufacturing under a stringent GMP regulatory framework, which is critical for commercial-scale GMP manufacturing. Closing and automating the entire processes produce consistent product quality and reduce the risk of contamination during each step of the workflow, enabling a significant cost reduction and ensuring regulatory compliance through standardized manufacturing and process reproducibility (James, 2017; Moutsatsou et al., 2019).

Closing the process is often achieved by using SUTs, which protect against contaminants outside of a cleanroom environment or biosafety cabinet. SUTs include disposable tubings, connectors, bags for cryopreservation, bioreactors and product transfer, vials, mixers, and filters. Closed system connectivity for sterile fluid transfer is accomplished through the use of tube welding/sealing or aseptic connectors. Tubing used in cell therapy manufacturing comes in different sizes (i.e., 1/8′′ ID, 1/4′′ ID, and 3/8′′ ID) and materials (i.e., PVC and C-Flex^®^). Tube welding/sealing is a widely adopted method for sterile connections in biopharma industries because of its ease of use. The most commonly used tube welders are Terumo TSCD^®^ II, Terumo SCB^®^ IIB, and BioWelder^®^ Total Containment from Sartorius. However, some inherent challenges remain such as particulate generation during the welding process, inability to join tubing of different sizes, different welders required for different tube sizes, and different types of thermoplastic tubings cannot be welded together (Clarke et al., 2016). To address these challenges, aseptic genderless connectors would offer the flexibility to work with any tubing size or material. Genderless connectors utilize three simple steps to enable sterile connection, “flip-click-pull” (CPC AseptiQuik^®^) or “click-pull-twist” (Pall Kleenpak^®^ Presto). These aseptic connectors are however not pre-fitted with tubing. Cell therapy manufacturers would have to work with their suppliers to customize single-use bags with compatible tubing dimensions for sterile connections or welding to other SUTs.

Automation is critical for large-scale commercial GMP manufacturing and most importantly enables closed system processing (James, 2017). There are two types of closed automation platforms for cell therapy manufacturing: (1) Closed automated system with integrated incubation and (2) Closed automated system with centralized incubation (James, 2017; Ball et al., 2018). In option 1, all of the unit operation processes are combined into a single automation system (e.g., Miltenyi’s Prodigy and Lonza’s Cocoon) and are specifically designed for autologous cell therapy manufacturing. These devices can do parallel processing in Class C processing area with minimal labor, “but” the processing equipment is poorly utilized due to the lengthy incubation periods, e.g., one machine processes one patient at a time and it is locked for use for 1–2 weeks depending on the number of cells that are expanded. This would result in the need for more machines and increases the cost-of-goods (COGS) for processing more patients for a given duration (James, 2017; Ball et al., 2018). Also, scalability for different MSC batch sizes due to the limited incubator space is one of the major challenges (Nguyen, 2016) since multiple dosing might be required for MSC therapy due to limited engraftment and survival rate of transplanted cells (Wysoczynski et al., 2018; Pittenger et al., 2019). In contrast, option 2 provides end-to-end manufacturing by integrating different modular automated systems and is highly suited for both autologous and allogenic manufacturing. This modular approach allows parallel processing in Class C area with high equipment and facility utilization achieved by separating incubation. It provides process flexibility for optimizing different conditions and the ability to incorporate new technologies that are critical for the early stage translational therapy developers (Ball et al., 2018). However, the modular approach requires careful selection of automation systems for physical and digital integration of different unit operations.

### Existing and Proposed Closed Automation Solutions for Each of the Unit Operations

#### Cell Isolation

Cell isolation is the first unit operation in hMSC manufacturing. The most common sources of hMSCs are bone marrow (BM), adipose tissue (AT), placenta (P), and umbilical cord (UC). In current clinical trials, bone marrow is the most widely used source of hMSCs followed by umbilical cord, adipose, and placenta (Pittenger et al., 2019). Currently, most of the cell isolation methods are manual or semi-automated followed by plating in either multiple T75/T175 cell culture flasks or cell stacks/cell factories depending on the starting cell numbers. Subsequently, the attached cells are harvested and cryopreserved as MCBs following standard critical quality attributes (CQA) testing. Furthermore, MCBs undergo a series of seed trains where post-expanded cells at the right passage are harvested and cryopreserved as working cell banks after CQA evaluation. Based on the CQAs, identifying the maximum passage limit with the same clinical efficacy as the earlier passages for each cell line is very important. This would identify the number of passage expansions and working cell banks required to achieve the maximal number of doses from a single vial of MCB. Depending on the starting hMSC source, there are potential gaps in the isolation methods that need to be addressed before adopting the entire workflow in a GMP facility.

Typically for BM-MSCs isolation, manual Ficoll-based density gradient centrifugation of bone marrow aspirates is carried out to separate the mononuclear cell (MNC) fraction. Compared to manual MNC separation, automated Ficoll-based density gradient centrifugation devices such as Sepax C-Pro (Cytiva) can be used to generate clinical-grade MSCs from human bone marrow or cord blood with high recovery and less processing time (Aktas et al., 2008, 2010; Hanley et al., 2013).

Conventionally, UC-MSCs are isolated from sections of perivascular tissue by two manual methods: (1) Direct explant culture technique. Although the procedure is straightforward, there are many challenging steps and contamination risks involved in handling the samples, and it is difficult to translate into an automation platform (Beeravolu et al., 2017). (2) Using a combination of mechanical dissociation with enzymatic degradation followed by filtration of undigested particles and direct culture of separated cell suspension (Smith et al., 2016). In line with the second method, a semi-automated approach of placental MSC isolation was carried out using sterile paddle blender bags containing pieces of intact placenta along with a cocktail of digestive enzymes. The semi-automated process yields were comparable while more importantly the processing time was significantly reduced to 1.5 h against 4–5 h using the manual method (Timmins et al., 2012).

To isolate AD-MSCs, lipoaspirates or adipose tissue are mixed with an equal volume of saline; subsequent manual centrifugation of the lipid phase aspirates is enzymatically digested using collagenase followed by manual centrifugation to isolate stromal vascular fraction (SVF). Alternatively, without the need for enzymatic digestion, rapid isolation of AD-MSCs using the blood-saline portion of lipoaspirate was demonstrated through a simple five-step process (Francis et al., 2010). Güven et al. (2012) previously reported an automated procedure to isolate AD-MSCs from adult human lipoaspirates using Sepax C-Pro, and compared to manual separation, automation resulted in a 62% higher isolation yield and a 24% higher frequency of clonogenic progenitors. More recently, Rodriguez et al. (2017) reported that SVF isolated from adipose tissue using three semi-automated medical devices (GID SVF-1^TM^, Puregraft^TM^, and Stem.pras^®^) are equivalent to the reference manual method in terms of SVF yield, characteristics, and clonogenic potential.

**FIGURE 1:**
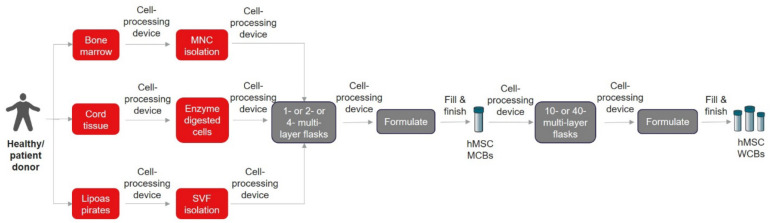
Closed and automated cell isolation workflow. The list of cell processing devices and fill and finish instruments are shown in Tables 2, 3, respectively.

Alternatively, other automation devices such as spinning membrane-based filtration device (LOVO^TM^, Fresenius) (Wegener, 2014) and cell separation based on size using a counterflow centrifugation system (Gibco^TM^ CTS^TM^ Rotea^TM^, Thermo Fisher Scientific) (Li et al., 2019; Dargitz et al., 2020) could be explored for non-Ficoll-based MNC isolation (BM-MSC), SVF wash and isolation (AD-MSC), and cord-tissue processing (UC-MSC) using the cited protocols. One other gap that needs to be highlighted here is the requirement and cost of different GMP-grade enzymes for digesting tissues from different sources. Interestingly, these alternatives or new-to-the-market bench-top closed automated cell processing systems could open more innovative ways to improve or simplify the existing protocols to maximize the isolation efficiency. Overall, we envision that the cell isolation process can be closed and automated as illustrated in Figure 1.

#### Cell Expansion, Processing, and Formulation

**FIGURE 2:**
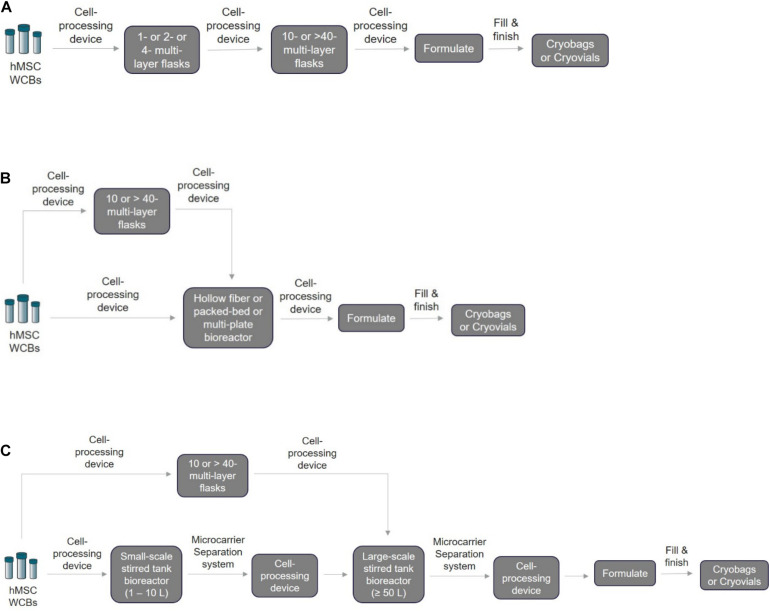
Schematic of the closed and automated hMSC manufacturing workflow. For the three methods listed below, the first step is to thaw the cryopreserved WCBs based on the required seeding density using a cell processing device to wash away cryopreserved media and exchange it with fresh expansion media. Depending on the technology chosen, it may be possible for the same cell processing device to be used for volume reduction, harvesting, passaging, and final formulation into bags for fill and finish. **(A)** 2D monolayer scale-out expansion using multi-layer systems. Processed cells can be transferred directly to a multi-layered flask for expansion. Next, the expanded cells after harvesting and volume reduction can be passaged directly in larger flasks until final harvest and formulation. **(B)** Scale-out expansion using hollow fiber membranes/multi-plate/packed-bed bioreactors. Typically, for hMSC manufacturing using the HF, MP, or PB bioreactors, two approaches can be adopted depending on the passage limit: (1) direct cell seeding into these bioreactors for cell expansion or (2) cell seeding into a 2D monolayer multi-layer device first before seeding into bioreactors. **(C)** Scale-up using microcarrier-based suspension culture in stirred-tank bioreactors. For this technology, two different closed and automated scale-up approaches can be used after cryopreserved WBCs are processed: (1) 2D–3D expansion: cells can be seeded and expanded first into 2D monolayer multi-layered flasks followed by seeding and expansion in 3D microcarrier-based suspension bioreactors. (2) 3D–3D expansion: cells can be seeded directly on 3D microcarriers for expansion in small-scale stirred tank bioreactor followed by seeding and expansion in large-scale stirred tank bioreactors. For in-line separation of microcarriers and cells, closed single-use systems such as the Harvestainer microcarrier separation system can be used for both smaller-scale and larger-scale applications.

Based on the ongoing clinical trial data, hMSCs are transfused intravenously at typical doses of 1–2 million cells/kg and in few cases not exceeding more than 12 million cells/kg (Galipeau and Sensébé, 2018), which is approximately 100–150 million cells/patient (Kabat et al., 2020). In addition, depending on the disease indications, the estimated hMSC dosage per patient might be from 15 million to 6 billion cells (Chen et al., 2013). Choosing a suitable scale-out or scale-up strategy for autologous and allogenic hMSC manufacturing (Figure 2) is critical to plan right at the beginning stage of small-scale process optimization. Depending on the scale needed, the manufacturer must identify a suitable closed and automated cell processing system that can connect directly to multi-layered flasks and bioreactors to perform volume reduction, wash, medium exchange, and formulation (Table 2). Most of the academic developers and advanced cell therapy companies fail to address this aspect, causing profound risks such as increased time and costs to repeat clinical trials shadowed by re-optimization of entire process workflow (Tania et al., 2016; James, 2017). Specifically, MSCs expanded under different culture conditions such as 2D monolayer or 3D microcarrier-based suspension system have an impact on their biological properties and functions, making process and technology changes difficult after clinical trials begin (Cherian et al., 2020; Levy et al., 2020).

**TABLE 2:** Closed automation cell-processing instruments that are commercially available in the market.

Supplier	Cytiva	Cytiva	Sartorius	Fresenius	Thermo Fisher Scientific
**Product specifications**	**Sefia S-2000**	**Sepax C-Pro**	**kSep400**	**Lovo**	**Rotea**
Output volume (ml)	15–400	8–500 (Optimal output recommended is 70 ml)	>50	50–50000	≥5
Technology	Syringe chamber centrifugation	Electric centrifugation motor and pneumatic circuitry for piston drive	Counterflow centrifugation system	Spinning membrane filtration	Counterflow centrifugation system
Scalability	<10 L	20–1200 ml	0.05–500 L, cell capacity per cycle (1–80 × 10^9^ cells)	Up to 22 L	0.03–20 L (no maximum volume, continuous processing possible), cell capacity per cycle (5 × 10^7^–5 × 10^9^ cells)
Versatility (applications)	Cell isolation by density gradient separation, harvest and formulation	Cell isolation by density gradient separation, spinoculation, harvest wash, and formulation	Wash, concentrate, and harvest	Fresh, cryo-preserved, and culture-expanded white blood cells, including, but not limited to, leukapheresis CD34 + cells, CAR T-cells, TILs, NK cells, and MSCs	Cell isolation and separation based on size, RBC depletion/lysis, fresh/cryopreserved/culture-expanded immune cells (CAR T cells, NK cells), MSCs, HEK, iPSC spheroids wash and concentrate, media exchange, harvest, and formulation
Key features	Ultrasound sensors for bubble detection, pressure and bag weight sensors, centrifugation up to 1600 *g*, Thermal mixer for temperature control between + 4°C and + 40°C and Separation chamber temperature monitor and control	Pressure monitoring and optical line sensors, centrifugation 100–800 *g*	3/8′′ × 1/4′′ C-Flex connections. Max g-force 1000 × *g*, max flow rates (1900 ml/min)	Membranes have 4-μm pores, using the Lovo Software 3.0. Multiple Source container processing, Administrator ability to pre-fill and lock operator entry fields and options	Instrument (Kit Barcode Reader, Bubble Sensors, pinch valves, camera, moisture sensor, Chamber Detector, OD and Pressure sensors), max g-force 3000 × *g*, max flow rates (165 ml/min) and Single-use kit (Bubble Trap, flexible 1/8-inch tube input (7) and output (1) lines, CFC Chamber) and Software (protocol builder with a simulator, process model and GUI). Able to connect to 2D and 3D expansion vessels. Visually monitor the cells in real time using Gibco^TM^ CellCam^TM^ video technology
Customization (consumables and protocol)	Two different protocol software’s and two different kits	Seven different protocol software’s and three different kits	One single-use class VI product. One software for all systems	Up to 10 protocols can be saved on the device and each wash cycle may be customized even further	One standard single-use kit (standard/high-flow version). More than 10 standard protocols for different applications. Protocols are highly customizable during process optimization. During GMP manufacturing software allows lockdown of protocol and restricts user access.
Dimensions (L × W × H); weight	51 × 74 × 91; 40 kg	40 × 27 × 46 cm; 16.3 kg	107.5 × 72 × 140 cm; 350 kg	45.7 × 50.8 × 67.3; 34 kg	29 × 50.8 × 76.2 cm; 20 kg
Translate to GMP	Yes (traceability using Barcode reader Data management with PDF reports)	Yes (traceability using Barcode reader Data management with PDF reports)	Yes	Yes (Exportable from DXT to Excel or LIMS)	Yes (OPC-UA interface to connect to a DCS, MES or 21 CFR Part 11–compliant system, digital integration using Delta V platform)

2D monolayer-based scale-out culture system:

Currently, for large-scale manufacturing of hMSCs in 2D monolayer cultures, “scale-out” expansion is carried out using multi-layered cell stackers (Corning^®^ CellSTACK and Nunc^TM^ Cell Factory^TM^) through 1 layer to 40 layers. Mostly, these systems are equipped with dual ports closed by caps for filling and venting, respectively. Thus, for aseptic processing, these systems require a laminar hood for manual handling, which is not ideal for large-scale manufacturing. However, it can be completely closed by having a 0.2-μm pore, hydrophobic membrane filter in one of the ports to allow gas exchange without the risk of contamination, while the second port can be internally sealed using an aseptic connector that can be connected to media bags or cell processing instruments to allow direct fluid transfer via pumping or gravity. Figure 2A shows the schematic for a closed and automated 2D monolayer-based two passage, scale-out hMSC manufacturing. Although this method (Figure 2A) is the most cost-effective (Mizukami et al., 2018) and a preferred option for expanding hMSCs for clinical trials (Rowley et al., 2012), many bottlenecks still exist. It is not a scalable system as it requires a larger footprint for handling due to its restrictive surface-to-volume ratio, its non-homogenous expansion due to non-uniform surface coating resulting in batch-to-batch variability, it being laborious, and the fact that media exchange and cell harvesting can be impacted by the handling of multiple stackers at the same time (Cherian et al., 2020). Alternatively, GMP-compliant, closed automated and single-use systems may be suitable for hMSC manufacturing including hollow fiber-based (HF) continuous perfusion device, the Quantum cell expansion system from Terumo BCT (Hanley et al., 2014; Barckhausen et al., 2016; Khan et al., 2017; Frank et al., 2019; Vymetalova et al., 2020), 2D multiplate-based (MP) Xpansion^®^ bioreactor system from Pall corporation (Rouard et al., 2020), and packed-bed (PB) iCeLLis^®^ bioreactor from Pall corporation (Mizukami et al., 2018) (Figure 2B). Nonetheless, all of these systems (HF, MP, and PB) are better suited for autologous therapy or scale-out allogeneic therapy as they are limited by scalability, poor harvesting efficiency (especially PB), and the least cost-effective technology (Mizukami et al., 2018).

3D microcarrier-based scale-up suspension system:

On the contrary, microcarrier-based suspension culture using stirred tank bioreactors provides a high surface-to-volume ratio, enabling high-density cultures for large-scale allogenic hMSC manufacturing (Schnitzler et al., 2016). More importantly, microcarrier cultures are the most cost-effective in terms of COG/dose closely following 2D monolayer cultures or even surpassing if we optimize the harvesting efficiency (Mizukami et al., 2018). Many GMP-grade commercial microcarriers are available: cross-linked dextran-based Cytodex^®^ 1 and 3 (Cytiva), SoloHill^®^ polystyrene (Sartorius Stedim), and untreated or Synthemax II or CellBind-coated polystyrene (Corning^®^). On the single-use stirred-tank bioreactors, there are a variety of commercially available options (Mobius^®^ from EMD Millipore, CelliGen^®^ from Eppendorf, BIOSTAT^®^ from Sartorius, HyPerforma^TM^ from Thermo Fisher Scientific, Xcellerex^®^ from Cytiva, and Allegro^®^ from Pall) for different scales starting from bench-top (1–5 L) and large pilot scale (10–300 L) bioreactors (Schnitzler et al., 2016; Jossen et al., 2018). This microcarrier-based technology also has limitations, such as non-uniform binding during cell attachment, poor harvesting efficiency (∼60%) (Mizukami et al., 2018), and the need for an additional step to separate microcarriers and cells post-harvest. Thus, small-scale process optimization by pre-screening different microcarriers with different substrate coatings in the media of choice using shake flask or spinner flask is important to identify the top-performing microcarrier and further optimize attachment, expansion, and harvest parameters (aeration, impeller speed, and feeding regime) for the large-scale transition. To date, many reports have shown successful scale-up of hMSCs in small-scale and large-scale bioreactors (Timmins et al., 2012; Schirmaier et al., 2014; Cunha et al., 2017; Lawson et al., 2017; Bayne et al., 2019; Koh et al., 2020). In Figure 2C, we have shown the schematic of closed and automated hMSC manufacturing using both 2D to 3D and 3D to 3D expansion methods.

#### Fill and Finish

As a last step in the entire manufacturing workflow, closed automated fill and finish is one of the most important cell therapy manufacturing processes primarily to ensure product safety, consistency, and integrity for longer-term storage (Brandon Fletcher et al., 2020). As such, cell therapy manufacturers need to be aware of the choice of equipment and the aseptic containers used for filling their products in a sterile and scalable manner. The most commonly used forms of containers are closed cryovials or cryobags. Some commercially available cryobags are multiple-chamber CryoStore^TM^ freezing bags (Origen), CryoMACS^®^ Freezing Bags (Miltenyi Biotec), and Freeze-Pak^TM^ Bio-Containers (CharterMedical) with different fill volumes. Gao et al. (2019) reported that human AD-MSCs cryopreserved at -150°C for 24 months in cryobags post-thaw had a viability of >90% with minimal cell clumps with functionality profiles similar to fresh cells. In the case of cryovials, it has been reported that the seal integrity could be compromised for glass vials with rubber stoppers at cryogenic temperatures thus presenting problems of losing product integrity during the critical cryopreservation stage (Zuleger et al., 2012; Hunt, 2019). Alternatively, CellSeal^®^ cryovial (Sexton Biotechnologies) made from USP Class VI material was reported to be stable and durable after 12 weeks of storing cryopreserved regulatory T cells with high cell recovery post-thaw (Fearnot et al., 2014). Aseptic technologies’ ready-to-fill AT-Closed Vial^®^ is made up of a polymer body with a thermoplastic septum, and the filling process is simple and scalable (Hunt, 2019). Pharmaceutical-grade Daiko-Crystal Zenith plastic vial was found to be suitable for hMSC cryopreservation at either -85 or -196°C for 6 months, with post-thaw viability of >95% with comparable growth and differentiation profiles of fresh controls (Woods et al., 2010). Ideally, cryobags are used for large-volume and take a longer time for thawing, while the cryovials can be thawed rapidly because of lower fill volumes. For both cryobags and cryovials, slow freezing and rapid thawing are critical to protect the structural and functional integrity of the cells (Hunt, 2019). Overall, examples of commercially available fill and finish instruments are shown in Table 3. As listed, very few systems are available that have been designed to suit the specific needs of cell therapy. The challenge for manufacturers is to choose an optimal system, cryopreservation media, and containers that will suit their current needs but be scalable as they progress through the clinical trials and into commercial production.

**TABLE 3:** Closed automated fill and finish instruments.

Specifications	Sexton CellSeal AF-500^TM^	Sexton Signata^TM^ CT-5	Terumo Finia	Flexicon FPC-50	Invetech’s 3rd gen	Aseptic technologies L1 robot
Containers	Vials	Bags and vials	Bags	Vials	Bags	AT-Closed Vials
Fill accuracy	99%	N/A	±2 ml or ±10% of the target volume, whichever is greater	±0.5% > 1 ml and ± 1% > 0.2 ml	N/A	N/A
Fill volume	0.8–5 ml	Up to 1500 ml	20–174 ml	<0.2–100 ml	0.25–5 L	0.1–50 ml
Fill capacity	400 vials/h	1 ml to 400 ml/min	N/A	1500 vials/h	N/A	600 vials/h
Batch size	N/A	N/A	N/A	N/A	N/A	≥100–5000 vials per shift
Sterilization	Vapor Hydrogen Peroxide (VHP)	Single-use kits	Single-use gamma-sterilized functionally closed tubing sets	Single-use fluid path	Single-use kit	Vapor Hydrogen Peroxide (VHP)
GMP compatibility	Yes (traceability of process parameters that is automatically generated in batch records and audit trials)	Yes (lockdown GMP compliant routines)	Yes (data management capability tools allow monitoring the processing run data and tracking accessibility)	Yes (able to generate batch reports after each production run and it comes up with optional software to support 21CFR Part 11)	Yes (21CFR11 compliant, eBR integrated)	Yes
Temperature control	External	External	Yes	N/A	N/A	N/A
Dimensions (L′′ × W′′ × H′′)	19.7 × 46.3 × 20	N/A	19.6 × 35 × 30.9	53.8 × 21.7 × 27.2	N/A	47 × 30 × 37
Additional features	The machine is designed for a controlled environment in both Class B and C cleanrooms that includes a benchtop, biosafety cabinet, or isolators.	The system is flexible with ready-to-use consumable kits that can be connected to run custom routines or optimized protocols. Able to perform, cell wash/media exchange and media preparation.	The system can maintain final product temperature to within 3°C, cell viability of more than 95%, uniformity of cell concentrations to within 5% for all bags	The equipment is designed for use under a biosafety cabinet or in a restricted access barrier system or customized for integration into an isolator.	Can perform bulk media formulation of 50–250 L	No need for Class B room

### Digital Integration of Different Unit Operations

While we can physically integrate modular closed and automation systems for each of the unit operations, enabling digital integration using software platforms provides true automation across the entire autologous or allogenic hMSC end-to-end manufacturing workflow. In this complex environment, data logging the information through enterprise resource management (ERP) starting from sourcing of the raw materials, manufacturing process controls, quality management through to product storage and delivery to the patient is critical. This will ensure a proper flow of information ensuring traceability, which is a requirement for a GMP manufacturing. Building this foundation of cell therapy digital integration and data management enables the interaction of production (hardware and controllers) and control layers such as supervisory control and data acquisition (SCADA) and manufacturing execution systems (MES). Moreover, the enterprise layer will facilitate interlinking the process and plant control for managing all aspects of clinical manufacturing. Data mining tools allow for the acquisition of upstream and downstream process batch record data and using this; real-time data analysis can be performed for different batches for rapid process optimization and troubleshooting. There are few automation transformation platforms that hMSC clinical manufacturers could leverage for integrating all bioprocess unit operations in a GMP biomanufacturing capacity (Supplementary Table 2).

Taken together, it is extremely important to understand the existing process, COGS, and choose the best closed automation technologies suitable for unit operations that will seamlessly scale and transition to GMP manufacturing. For example, the hMSC manufacturers, depending on the scale, could choose cell stackers, or stirred tank bioreactors, etc. for incubation. Cell processing devices (Table 2), can be utilized for cell isolation, concentrating, washing cells, formulation, and then fill and finish into vials or bags using appropriate instrumentation (Table 3) that suits the preferred product format and number of doses. Note that cell processing systems will be used for multiple steps in the entire manufacturing workflow (Figures 1, 2) and the manufacturers need to evaluate each system individually to identify the best fit that works for their process. During this early stage evaluation, it is also important to qualify tubing compatibility and connector options to integrate instruments from several suppliers. Overall, hMSC manufacturers need to embrace the idea to design a GMP facility capable of flexible manufacturing using closed modular systems compatible with a digital connectivity platform to enable fully automated manufacturing.

## Characterization and Safety Testing to Ensure Product Consistency Throughout the Manufacturing Workflow

During each step of the unit operations starting from cell isolation through to final product cryopreservation and ensuing patient delivery, it is imperative that the cell product undergoes QC and monitoring, such as critical process parameters and critical material attributes, to ensure that CQAs are met for the end product, thereby maintaining product consistency in terms of quality and performance.

Since the late 1960s, the US FDA has been establishing GMP guidelines for pharmaceuticals and has been periodically updating them (Immel, 2001). While those are useful for small-molecule drugs, it is almost impossible to apply those rules directly to our “living” drugs as we do not have a complete understanding of (1) the cells we work with and (2) the intricacy of the manufacturing process. Hence, regulators have developed new guidance with regular periodical updates for cell therapies.

The mandatory CQAs for characterization include identity, purity, potency, and safety. Table 4 summarizes the main framework and requirements necessary for characterization testing. Of note, a review in 2017 observed that a substantial proportion of hMSCs trials did not result in publications; in particular, early stage trials were unsuccessful (Fung et al., 2017). Of those that did report their findings, a sizable number reported a lack of potency as a reason for not advancing their candidates (Fung et al., 2017). More importantly, manufacturing and non-manufacturing variables have been put forward as the possible cause for the discrepancy between previously observed efficacy in both *In vivo* and *In vitro* settings that differed from the clinical settings (Galipeau and Sensébé, 2018). An underlying reason for this is the inability to accurately predict critical hMSCs functions, such as its immunomodulatory activity properly define CQAs.

**TABLE 4:** Characterization/CQA for MSC therapy.

	Identity	Potency	Purity	Safety
Definition and Purpose	21 CFR 610.14: Specific testing that will adequately identify the designated product and distinguish it from any other product being processed in the same site. Also, to ensure that the final product given to the patient is as intended and that the manufacturing process did not significantly alter the starting hMSCs (FDA, 1998a).	21 CFR 610.10: Potency assays are necessary to quantify specific hMSCs biological functions for the intended purpose (FDA, 2011).	21 CFR 600.13: To define that the final product is relatively free from any extraneous material (FDA, 2008).	21 CFR 610. 12: To ensure that the product is free from any adventitious agents and other contaminants (FDA, 2008).
Key consideration	Multiple factors such as culture duration and scaling up could result in changes to the final hMSCs product. The US FDA notes four major parameters that can affect MSCs characteristics (Mendicino et al., 2014): Working and Master cell bank Fetal bovine serum (FBS) Oxygen concentration Cryopreservation	A matrix of relevant assays that likely demonstrates the mechanism of action (MOA) of hMSCs for the intended purpose rather than a single assay should be considered. Given that hMSCs have multiple clinical indications, there is no established gold standard potency assay. Guidance from the US FDA recommends how to establish potency assays, and that they be performed during the early product development phase due to the large number of advantages it affords (FDA, 2011).	Broadly classified as two groups, pyrogenicity/endotoxin, and residual contaminants	Required testing includes adventitious viruses and sterility testing of bacterial and fungal and mycoplasma (FDA, 2008). US FDA recommends reading of two guidance document [“Points to Consider in the Characterization of Cell Lines Used to Produce Biologicals” (FDA, 1993) and ICH guidance Q5A: “Guidance on Viral Safety Evaluation of Biotechnology Products Derived From Cell Lines of Human or Animal Origin (FDA, 1998b)]
Requirements	No specific assay is stated; however, it is a requirement to confirm the identity of the cells via quantitative testing through phenotypic and/or biochemical assays such that it can be adequately identified and distinguished from other products (FDA, 1998a).	No specific assay is stated, however, to attain a biologics license, the hMSC product would have to meet the requirements of potency as stated in (21 CFR 601.2), which requires the validation of a potency assay “accuracy, sensitivity, specificity, and reproducibility” [21 CFR 211.165(e)]. Additionally, data from all tests, with the necessary standards and specifications, must be well documented (21 CFR 211.194) (FDA, 2011).	*Pyrogenicity/endotoxin:*(FDA, 2008) 21 CFR 610.13: Requires the rabbit pyrogen test method. If this test cannot be carried out, the Limulus Amebocyte Lysate test method (LAL) is an alternative, only if acceptable conditions as set by the FDA guidelines are met. US FDA recommends an upper limit for endotoxin at 5 EU/kg body weight/h, provided it is not administered intrathecally (upper limit would be reduced to 0.2 EU/kg body weight/h if so) *Residual contaminants:*(FDA, 1998a) Parameters include: Residual peptides and proteins used during production and purification Manufacturing reagents, such as cytokines, growth factors, antibodies, beads, and serum. Quantification of cell debris or contaminating cell type	*Adventitious agents:* (FDA, 1993; FDA, 1998a) As per 21 CFR 610.12 *In Vitro* and *In Vivo* viral testing is necessary. Key information required includes what are the tests performed and at which stage of the manufacturing process was it done. Species-specific testing for adventitious viruses is also important. With human cells, it is recommended that human pathogens such as CMV, HIV-1 and 2, HTLV-1 and 2, EBV, HBV, HCV, and B19 be tested. *Sterility testing:* (FDA, 1998a) Specific microbiological tests are described in 21 CFR 610.12 culture and non-culture-based methods. *Mycoplasma testing:*(FDA, 1993; FDA, 1998a) Both culture-based assays and polymerase chain reaction-based assays can be used.

Expectedly, much focus has been given to identity, purity, and potency, particularly during process development. The aspect of safety has been thought to be a step considered only at the final stage of manufacturing. However, to ensure that the final hMSC product is free of any adventitious agents, safety should be considered at each stage of the manufacturing process, starting from the selection of raw material as previously mentioned. Additionally, it is important to note that safety testing has three separate components, namely, sterility, mycoplasma, and adventitious virus testing that must be completed (Guadix et al., 2019).

In this section, we describe what are the best practices as per literature in terms of hMSC characterization testing, and what should possibly be considered to overcome the current challenges in product quality assessment. Moreover, we suggest additional considerations that are being considered by the field that would improve hMSC product efficacy with regard to different clinical indications.

### Established Technology Parameters

While certain methods have been well established, it does not mean that they can be used directly across multiple utilities due to key differences between them. For example, flow cytometry assay for cellular identity is well accepted and multiple parameters still should be considered. These include but are not limited to the need for a reference control for specific cell surface markers and to overcome the subjective nature of the instrument type and operator gating strategies. Interestingly, standardized automated gating has been shown to improve the precision of the assay, which can be included as an established method (Suni et al., 2003).

### Quantification of Multiple Parameters in a Single Assay

The immunomodulatory functions of hMSCs via the release of cytokines have been demonstrated to be a key mechanism in which they are utilized for the treatment of various clinical indications. This point has been highlighted in multiple reviews, which summarize the plethora of cytokines that have been demonstrated to be released by hMSCs, as well as their downstream regulatory functions (Kyurkchiev et al., 2014; Gao et al., 2016; Han et al., 2019). As such, it is critical to characterize the hMSC cytokine release profile and expression levels, to demonstrate potency and consistency in manufacturing.

The testing of multiple cytokines individually for hMSCs would be costly, time-consuming, and ultimately impractical. However, the need for comprehensive characterization testing should not be a trade- off given its importance. As such, assays such as the multiplexed ELISA to test cytokine and growth factors residue for potency and purity are being considered (Ellington et al., 2010; Tighe et al., 2015). The attractiveness of this platform is that it allows for the concurrent quantification of multiple cytokines, at a fraction of the cost, relative to testing cytokines individually.

Of note, while this platform has yet to be fully adopted in approved hMSCs therapy, its feasibility, however, has been shown in other approved therapeutics. Recent examples include the vaccine development for the Sars-Cov-2 pandemic. As part of BioNTech-Pfizer COVID-19 RNA vaccine, a Luminex assay was used (the ProcartaPlex) to test 11 different T_*H*_1 and T_*H*_2 cytokines concurrently (Sahin et al., 2020). Another example was the development of Axicabtagene Ciloleucel. This CAR T-Cell Therapy for Refractory Large B-Cell Lymphoma employed a multiplex Luminex assay to measure seven different cytokines during their process development stage (Turtle et al., 2016). Hence, this is a platform that could be adopted for hMSCs as well. While some operational challenges are present, the benefits of such a platform warrant further looking into (Ellington et al., 2010; Tighe et al., 2015).

### Clinical Indication-Specific Assay

A best practice is to develop a matrix of relevant assays that likely demonstrates the mechanism of action (MOA) of the hMSCs for the disease indication for which the MSCs are intended. This is appropriate, given that hMSCs could have a specific effect on certain clinical indications. For example, in the treatment of graft-versus-host disease, hMSCs’ immunomodulatory capacity via anti-inflammatory cytokines should be part of the matrix of the potency assays (Caplan, 2009). However, should the same cell product be used for neural regeneration, whereby the hMSCs can stimulate angiogenesis in nerves and motor function recovery, other potency assays would be more relevant (Masgutov et al., 2019). As such, the selection of specific clinical indication potency assays would be more relevant in not only demonstrating the MOA of the cells but also reducing the need to perform other irrelevant potency assays and thus save the effort and time driving lower COGS in product development.

### When and Where Should Characterization Assay Be Done

The necessity of performing characterization assay is not solely because it is a requirement, rather, it is to help the developer ensure that the development and manufacturing process is consistent, and the final product will function as intended. The US FDA further recommends that characterization testing be done during the early product development phase due to a large number of advantages it affords such as the ability to “Demonstrate product activity, quality and consistency throughout product development; evaluating product stability; provide a basis for assessing manufacturing changes,” etc. (FDA, 2011). Hence, characterization testing should be performed as a part of in-process testing instead of just being part of the release criteria.

In addition to performing characterization assays early, it is also a good practice that such in-process testing should be done at key points of the manufacturing process, as well as critical risk areas for certain parameters. For example, when using frozen products, if it undergoes manipulation such as washing or culturing after thawing, it may be necessary to repeat the sterility test. This is more so if it is performed in open systems (FDA, 1998b). Moreover, concerning mycoplasma, two major sources in which contamination can occur are the culture facility: in particular open culture systems and the use of animal serum products (FDA, 1998a). It is recommended that mycoplasma testing be done at stages where cell pooling is involved to harvest cells or when there is an extended culture procedure (FDA, 1993).

### Other Consideration to Enhance the Development of MSC Therapy

#### Development of More Representative Characterization Assays: Direct Function Assays

One key challenge in the development of successful therapy is due to hMSC therapy being “living” drugs. As such, they are fundamentally a heterogeneous population, whereby their gene and protein expression profiles can be entirely different from each other. Several studies have demonstrated that this heterogeneity is due to multiple parameters, such as hMSC origin source (Hass et al., 2011), the method of extraction (Juneja et al., 2016), as well as *in vitro* culture conditions and methods (Yin et al., 2019), just to name a few.

As multiple parameters can affect hMSC functions, it stands to reason that more meaningful characterization assays should be employed (in addition to what is minimally needed) to certify that the final hMSC product has the necessary therapeutic activity (Chinnadurai et al., 2018). Indeed, more groups are looking to engineer more predictive characterization assays to ensure a greater correlation between the therapeutic activity and final product (Zhukareva et al., 2010; Chinnadurai et al., 2018; Russell et al., 2018). One such assay is the inhibition of activated CD4 + T-cells proliferation to measure hMSC immunomodulatory function. Given that this method allows for a phenotypic change to be measured, relative to surrogate measurements of cytokines such as TNF-α receptor expression, it is believed to be a more accurate representation (Bloom et al., 2015).

#### New Characterization Methods: Utilizing Genetic Data to Improve Clinical Indication Prediction

Given the decades of hMSC research and clinical trials, we have an enormous database of gene expression data that thoroughly investigates almost every aspect of the hMSC transcriptome throughout every stage of the manufacturing process. Hence, studying this readily available treasure trove of data is extremely useful to properly elucidate the nature of hMSCs, their role in multiple diseases, and potential clinical indication via its mechanism of action (Pittenger et al., 2019).

Indeed, one interesting transcription factor that was demonstrated to regulate hMSC effector function was Twist1 (Boregowda et al., 2016). Boregowda et al. (2016) observed that altering expression levels of Twist1 resulted in a corresponding change in hMSC potency in both *in vitro* and *in vivo* settings. Their team subsequently developed a CLinical Indications Prediction (CLIP) scale that could be used to prognosticate hMSC heterogeneity against hMSC effector function for multiple clinical indications. While this has the potential to be a useful tool for screening populations, more work should be done to establish it further.

#### Improving on Pre-existing Assays: Using New Assays to Speed Up Critical Characterization Parameters

There is a need to accelerate the development of hMSCs therapy; hence, there is much focus on improving existing protocols of characterization of CQAs (Samsonraj et al., 2017).

Concerning potency, as previously mentioned, the implementation of the multiplex ELISA to perform multiple cytokine measurements instead of single-target ELISA is being considered (Ellington et al., 2010; Tighe et al., 2015; Turtle et al., 2016; Sahin et al., 2020). For identity characterization, using gene expression assays such as Taqman^TM^ to replace standard Tri-lineage differentiation staining assay is another example. While the current Tri-lineage differentiation assay requires 2–3 weeks for the cells to differentiate followed by staining and imaging (Owston et al., 2019; Sanjurjo-Rodriguez et al., 2019; Rajpar and Barrett, 2020), newer methods such as the measurement of hMSC Tri-lineage gene expression following 1–2 weeks of differentiation reduces the time needed while providing quantitative information relative to the current method (Szepesi et al., 2016; Hwang et al., 2017; Ling et al., 2020).

Once properly validated, these new assays would be used as an alternative to current methods. One such example is the characterization of safety, in particular, mycoplasma detection. Traditionally, the detection of mycoplasma was done via culture methods that typically require around 28 days. However, it is now accepted by regulators that mycoplasma detection can be done via PCR-based assay, which is cheaper and less time-consuming than the traditional method (FDA, 2010).

#### Improving Manufacturing Processes

While developing better potency assays would allow the detection and subsequent removal of suboptimal hMSCs, it would be far better to develop techniques that indicate hMSCs’ therapeutic potential. This concept is not lost on several companies that had improved on the current manufacturing process.

One stage that was targeted was cryopreservation. A study showed that freshly thawed and washed hMSC had a large range in its viability (36–85%) (Matthay et al., 2019). This could be a result of cellular damage due to the cryopreservation stage in which a study observed that the process of cryopreservation affects the cytoskeleton (Chinnadurai et al., 2014). Of note, these detrimental changes to the hMSC physiology could lead to an increase in complement-mediated clearance (Moll et al., 2014), thereby significantly reducing its overall efficacy for the patient due to a decline in hMSC persistence in the system. In addition to viability and persistence, cryopreservation was also observed to reduce hMSC immunosuppressive function (Francois et al., 2012). Taken together, improving the cryopreservation process and characterizing the hMSC product before and after this stage is vital.

While this has not been fully validated, there is some evidence that provides credence to this strategy in improving MSC quality. Take the most recent approved hMSC therapy Alofisel^®^. During its phase 3 clinical trial, they took into consideration the pitfalls of cryopreservation by including an additional process. Before administration, they thaw the cells and formulated 120 million cells in 24 ml of culture medium before shipping to the respective hospital as a formulated product that can be stored for 48 h (Panes et al., 2016). This allowed “recovery” of freshly thawed product and selection of viable cells to occur, addressing the issue observed above. While this is promising, more studies would have to be done to further validate this strategy.

## Regulatory Perspectives of hMSC Products

Despite decades of research showing hMSC clinical potential and a vast number of hMSC clinical trials covering an array of indications, only a handful of hMSC products (Ancans, 2012) have been approved for market authorization globally compared to the large number of ongoing clinical trials that meet the safety requirements (Jahani et al., 2020). For country-specific information, the readers are directed to http://www.aabb.org/advocacy/regulatorygovernment/ct/international/Pages/default.aspx.

### Regulations for Mesenchymal Stromal Cell-Based Medicinal Products in the United States and European Union

**FIGURE 3:**
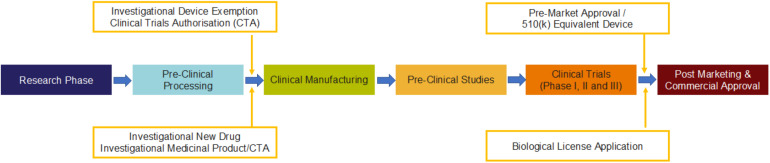
Process for the generation of MSC-based clinical product with compliance to regulatory requirements by FDA (Mendicino et al., 2019) in the cell and gene therapy category.

The process of approval for MSC-based clinical product is developed following a rigorous procedure designed in a sequential and stepwise manner as shown in Figure 3 in accordance with the global regulatory agencies (Table 5).

**TABLE 5:** Regulatory agencies in different countries.

Country	Regulatory agency	Website link
United States	Food and Drug Administration (FDA)	https://www.fda.gov/vaccines-blood-biologics/cellular-gene-therapy-products
Canada	Health Canada	https://www.canada.ca/en/health-canada/services/drugs-health-products/biologics-radiopharmaceuticals-genetic-therapies/applications-submissions.html
Europe	European Medicines Agency (EMA)	https://www.ema.europa.eu/en
China	National Medical Products Administration (NMPA)	https://www.emergobyul.com/resources/china/china-food-drug-administration
Japan	Pharmaceuticals and Medical Devices Agency PMDA	https://www.pmda.go.jp/english/
Korea	Pharmaceutical Affairs Act (PAA) Ministry of Food and Drug Safety (MFDS)	https://www.mfds.go.kr/eng/index.do
India	Central Drugs Standard Control Organization (CDSCO)	Central Drugs Standard Control Organization (CDSCO)
Australia	Therapeutic Goods Administration (TGA)	https://www.tga.gov.au
Germany	The Paul Ehrlich Institute	http://www.pei.de (not a regulatory site; but informative)

#### Research Phase

The most critical activity that happens is tissue procurement/donor qualification. The tissue procurement is done following Good tissue practices (GTP), using 21 CFR 1271^1^. It is intended to help manufacture human cells, tissues, and cellular and tissue-based products (HCT/Ps) and to comply with the comprehensive regulatory framework for HCT/Ps, outlined in Title 21 of the Code of Federal Regulations, Part 1271 (21 CFR Part 1271).

In Europe, the research phase mainly involves tissue procurement, and it is mainly done through EU Tissue and Cell Directives (EUTCD) following the Directive 2004/23/EC^2^.

#### Preclinical Processing

In this phase, in addition to 21 CFR 1271, additional rules of 361 PHS Act are applied. Under section 361 of the Public Health Service Act (42 U.S. Code §264), the U.S. Secretary of Health and Human Services is authorized to take measures to prevent the entry and spread of communicable diseases from foreign countries into the United States and between states.

In Europe, preclinical processing is carried out under the authority of EUTCD using the following directives: (i) Directive 2004/23/EC, (ii) Directive 2006/17/EC, and (iii) Directive 2006/86/EC.

#### Clinical Manufacturing

Once preclinical processing is approved, clinical manufacturing of cell doses is manufactured in good manufacturing practice (GMP) facility following 21 CFR 210 and 21 CFR211.

(a) 21 CFR Part 210 refers to Current Good Manufacturing Practice in Manufacturing Processing, Packing, or Holding of Drugs.
(b) 21 CFR Part 211 refers to Current Good Manufacturing Practice for Finished Pharmaceuticals.

In Europe, clinical manufacturing is also done in GMP facilities following the Directive 2003/94/EC.

#### Preclinical Studies

This is usually conducted in a good laboratory practice (GLP) facility using specifically approved animal models for a specific disease. Specific regulations governing this process are 21 CFR 58 and 21 CFR 610.

(a) 21 CFR Part 58 refers to GOOD LABORATORY PRACTICE FOR NONCLINICAL LABORATORY STUDIES.
(b) 21 CFR 610 refers to general biological products standards^3^.

In Europe, preclinical studies are done in GLP under (i) Directive 2004/9/EC and (ii) Directive 2004/10/EC.

#### Clinical Trials Phase I, Phase II, and Phase III

Once the data from preclinical studies are finalized, an investigational new drug (IND) application should be filed with the FDA before commencing a clinical trial. The IND application should include the clinical protocol and detailed descriptions of previous clinical experience, preclinical studies, manufacturing, and testing. These are done again in a sequential manner of Phase I (safety), Phase II (Efficacy), and Phase III (larger cohort).

(a) For IND filing, 21 CFR 312 is used. 21 CFR 312 refers to procedures and requirements governing the use of investigational new drugs, including procedures and requirements for the submission to, and review by, the Food and Drug Administration of investigational new drug applications (INDs). An investigational new drug for which an IND is in effect in accordance with this part is exempt from the premarketing approval requirements that are otherwise applicable and may be shipped lawfully to conduct clinical investigations of that drug.
(b) Besides, throughout all the phases of clinical trials, the following FDA regulations are applied:
  (i) 21 CFR 50—refers to the protection of human subjects
  (ii) 21 CFR 54—refers to the financial disclosure by clinical investigators
  (iii) 21 CFR 56—refers to the institutional review boards
  (iv) 21 CFR 11—refers to the electronic records and electronic signatures

In Europe, all clinical trials are done in IMP with the Directive 2001/20/EC and GCP with the Directives 2005/28/EC and Directive 95/46/EEC.

#### Post-marketing and Commercial Approval (Biological License Application, BLA)

Ensuring safety, efficacy, and success in a large cohort, the study moves to a final phase of commercial approval for use as standard care of therapy. For this to move into the final stage of therapy, the following regulations are applied.

(a) 21 CFR 600—refers to the regulations for biological products in general
(b) 21 CFR 601—refers to the applications for biological licenses; procedures for filing
(c) 451 PHS Act—refers to the natural resources and environmental protection act of public health safety

In Europe, Market authorization is done through (i) Directive 2001/83/EC, (ii) Directive 2009/120/EC, and (iii) Regulation EC 1394/2007 and Regulation EC 726/2004.

What is new with FDA regulations in 2020:

The FDA regulates the commercialization of cell therapy products through the Public Health Service Act (PHSA) of 1944 and the Food, Drug, and Cosmetic Act of 1938. FDA uses Sections 351 and 361 of PHSA to establish regulatory requirements for commercialization and safety for the human cells, tissues, and cellular and tissue-based products (HCT/Ps). For details of FDA administration of Section 361 versus Section 351 products, please refer to recent governmental regulations (Fang and Vangsness, 2020).

### Regulations for Mesenchymal Stromal Cell-Based Medicinal Products in Korea

Korea tops the list among the globe for the highest number of cell therapy- and gene therapy-approved products. The Korean Ministry of Food and Drug Safety (MFDS) is the regulatory body and uses their Pharmaceutical Affairs Act (PAA) for the regulations to govern the release or commercial launch of cell and gene therapy products^4^.

The process goes through just like the regulatory activities outlined in the figure for the United States, with minor modifications (Galli and Serabian, 2015). The process consists of three steps:

(a) Pre-IND meeting: this should have a prototype of the product developed in GLP in preclinical development.
(b) Application of IND: Consists and Phase I and Phase II clinical trials using a product manufactured in GMP.
(c) Submission of NDA: Following Phase III clinical trial, MFDS reviews (115 days) the data and enables NDA approval, following which the product is released into the market.

MFDS notifications include Regulations on Review and Authorization of Biological Products (RRABP) (Qiu et al., 2020). In the RRABP,

(a) Article 25: Safety and Efficacy Review Criteria^5^
  (i) Annex 2: types of information needed for cell therapy products
  (ii) Annex 3: information needed for gene therapy products
(b) Article 30: provides the specifications and test methods for cell therapy^6^
(c) Article 31: review criteria for gene therapy products^7^

## Conclusion

Although there are more than 900 hMSC clinical trials that are currently ongoing around the world, we have seen only limited success in product approvals for clinical therapy. A host of reasons go into the factor of why we see this trend, such as regulatory burdens due to high-risk raw material selection, failure to show product consistency and efficacy, cost of manufacturing, and open system processing. To overcome this, manufacturers need to start with the end goal in mind. First, choosing high-quality, low-risk raw materials with all the necessary QC/safety testing and regulatory support documentation is an utmost priority. Qualification of whether these raw materials can be supplied in a secure and scalable manner is essential to sustainability. Second, the evaluation of scalable closed and automated solutions must be done upfront during the early process development stage. Changes in scale and automation solutions are unavoidable because novel technologies will be developed in the future to address the current challenges. This process flexibility can only be adopted if the manufacturing workflow is modular in design. Third, it is empirical to test CQAs at every step of the manufacturing unit operations starting from the isolation step of hMSC, creation of MCBs and WCBs, to final product scale-up/scale-out expansion, before fill and finish and post-thawing of the cryopreserved product. Lastly and most importantly, there is no “one size fits all” approach for hMSC therapy regulatory guidance. Early and constant engagement with regulatory agencies to understand the relevant documentation required for multiple regions is requisite for smooth regulatory approvals. Taken together, planning ahead of time for a GMP regulatory-compliant manufacturing is key for successful hMSC-based therapies in the future.

## Author Contributions

PJ wrote the GMP-based closed and automated manufacturing section. RL contributed to the product characterization for MSC therapy. JN wrote the critical raw material quality assessment. MV covered the regulatory perspectives for MSC-based cell therapy products. All the authors, in addition to writing specific sections, reviewed and commented on the other sections. PJ and MV conceived the design, assembly of data, analysis, and interpretation.

## Conflict of Interest

PJ, RL, JN, and MV have compensated employment at Thermo Fisher Scientific.

## Footnotes

1 https://www.fda.gov/regulatory-information/search-fda-guidance-documents/regulation-human-cells-tissues-and-cellular-and-tissue-based-products-hctps-small-entity-compliance

2 https://eur-lex.europa.eu/LexUriServ/LexUriServ.do?uri=OJ:L:2004:102:0048:0058:en:PDF

3 https://www.gmp-compliance.org/guidemgr/files/CFR_2019/CFR-2019-title21-vol7-part610.pdf

4 https://www.mfds.go.kr/eng/index.do

5 https://www.mfds.go.kr/eng/brd/m_61/view.do?seq=46

6 https://www.mfds.go.kr/eng/brd/m_27/view.do?seq=70469

7 https://www.mfds.go.kr/eng/brd/m_61/view.do?seq=10

## Supplementary Material

Supplementary Table 1 Common hMSC culture systems, raw materials, and QC/Safety testing expectations.

Supplementary Table 2 List of different digital integration platforms.
