# Supplementary material for: Acceleration of Translational Mesenchymal Stromal Cell Therapy Through Consistent Quality GMP Manufacturing
Source: Front Cell Dev Biol. 2021 Apr 13;9:648472. doi: 10.3389/fcell.2021.648472 (PMC8076909; doi:10.3389/fcell.2021.648472)
Supplement: Supplementary Table 1 — Common hMSC culture systems, raw materials, and QC/Safety testing expectations. [file Data_Sheet_1.docx]

**Table S1: Common hMSC culture systems, raw materials, and QC/ Safety testing expectations**

| **Culture system** | **Animal serum based** | **Xeno-free hPL based** | **Serum- and Xeno-free** |
| --- | --- | --- | --- |
| Key supplements | FBS (2% - 20%) in basal media (DMEM, alpha-MEM) | hPL (2% - 10%) in basal media (DMEM, alpha-MEM) | Human serum albumin  Human transferrin  L-glutamine or GlutaMAX (freshly supplemented, AOF/ GMP-grade available)  Growth factors: Recombinant FGF2, TGF, PDGF, EGF, IGF |
| Passaging reagent | Trypsin-EDTA (bovine origin) – inactivation with serum required  TryPLE (AOF/ GMP-grade available) – inactivation not required. Enzyme is neutralized by dilution with buffer/ media | | |
| Substrate-coating requirement | Not required | Not required | Human Collagen  Recombinant Fibronectin  Affinity-purified human plasma fibronectin (Biological industries/ Cytiva)  rhVitronectin  rhLaminin-521 |
| Quality and Safety testing expectations on critical raw materials | - Viral clearance/ inactivation  -Sterility, endotoxin, mycoplasma.  Sourcing:  - Countries with Low TSE/BSE risks | Ref: Schallmoser et al 2019  Virus reduction:  -UVA, GI, S/D | -Sterility, endotoxin, mycoplasma.  - Viral clearance/ reduction (to the secondary level) |
| Relevant regulatory guidelines | Ph. Eur. 7th, Monograph 01/2008:2262 Bovine Serum  - USP <1024> Bovine serum  - USP <90> FBS quality attributes and functionality tests | Ref: Schallmoser et al 2019  - Ph. Eur.chapter 5.2.12 | Growth factors:  USP <92> Growth factors and cytokines used in cell therapy manufacturing |

Table S2: List of different digital integration platforms.

| Company | Digital Integration Tools | |
| --- | --- | --- |
| Cytiva® | Chronicle automation platform | A unified digital platform that provides electronic batch records and the ability to integrate third-party instruments |
| Thermo Fisher Scientific | TruBio discovery automation system | Based on the DeltaV platform (Emerson Technologies) and a distributed control system (DCS). Data can be easily stored and transferred during scale-up process optimization and commercialization. |
| Terumo BCT | StafaCT Suite | To support the entire cell therapy continuum including the capture of electronic product data and the manufacturing workflow. |
| Sartorius-Stedim | BioPAT® MFCS | Can be used for data management, automation, control, and monitoring under different modules. |
| Merck Millipore | BioContinuum™ Platform | Provides process and digital building blocks for continuous upstream and downstream bioprocessing coupled with data acquisition and analysis. |
